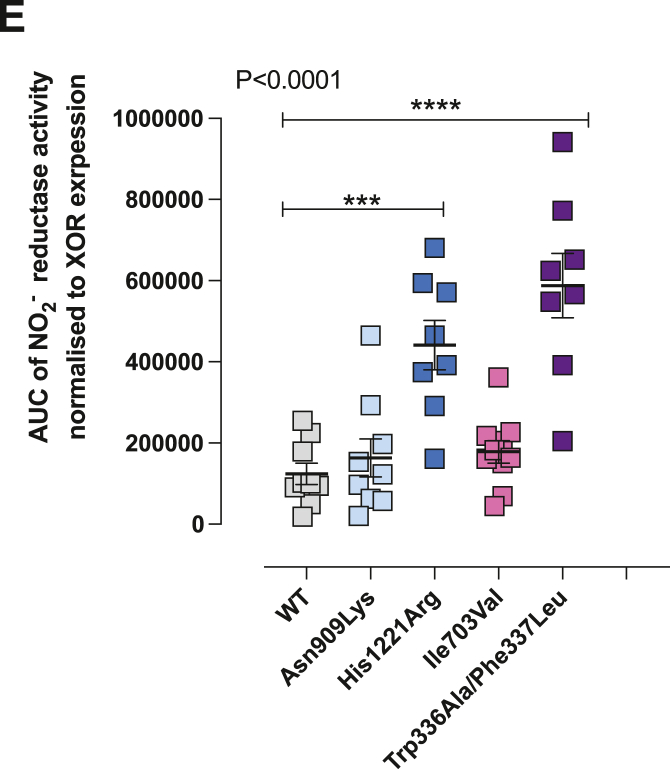# Corrigendum to “Natural mutations of human *XDH* promote the nitrite (NO_2_^−^)-reductase capacity of xanthine oxidoreductase: A novel mechanism to promote redox health?” [Redox Biol. 4 (67) (2023) 102864]

**DOI:** 10.1016/j.redox.2023.102925

**Published:** 2023-10-21

**Authors:** G. Massimo, R.S. Khambata, T. Chapman, K. Birchall, C. Raimondi, A. Shabbir, Nicki Dyson, K. Rathod, C. Borghi, A. Ahluwalia

**Affiliations:** a William Harvey Research Institute, Barts & the London Faculty of Medicine & Dentistry, Queen Mary University of London, Charterhouse Square, London, EC1M 6BQ, UK; b LifeArc, Accelerator Building Open Innovation Campus, Stevenage, SG1 2FX, UK; c Department of Medical and Surgical Sciences, Faculty of Medicine, University of Bologna, Via Massarenti, N.9, 40138, Italy

The authors regret an error in transcription for figure creation resulted in an incorrect representation of the data for Fig. 6E. The correct panel image is shown below. All data and statistical analysis statements described in the figure legend, visualised in the figure and discussed in the manuscript are unchanged.

The authors would like to apologise for any inconvenience caused.

**Figure:**